# Neural correlates of cocaine-induced conditioned place preference in the posterior cerebellar cortex

**DOI:** 10.3389/fnbeh.2023.1174189

**Published:** 2023-04-26

**Authors:** Olga Rodríguez-Borillo, Lorena Roselló-Jiménez, Julian Guarque-Chabrera, María Palau-Batet, Isis Gil-Miravet, Raúl Pastor, Marta Miquel, Laura Font

**Affiliations:** ^1^Área de Psicobiología, Universitat Jaume I, Castellón de la Plana, Spain; ^2^Dominick P. Purpura Department of Neuroscience, Albert Einstein College of Medicine, New York, NY, United States; ^3^Unitat Predepartamental de Medicina, Universitat Jaume I, Castellón de la Plana, Spain

**Keywords:** conditioned place preference (CPP), cerebellum, mPFC, nucleus accumbens, VTA, cFos, cocaine, addiction

## Abstract

**Introduction:**

Addictive drugs are potent neuropharmacological agents capable of inducing long-lasting changes in learning and memory neurocircuitry. With repeated use, contexts and cues associated with consumption can acquire motivational and reinforcing properties of abused drugs, triggering drug craving and relapse. Neuroplasticity underlying drug-induced memories takes place in prefrontal-limbic-striatal networks. Recent evidence suggests that the cerebellum is also involved in the circuitry responsible for drug-induced conditioning. In rodents, preference for cocaine-associated olfactory cues has been shown to correlate with increased activity at the apical part of the granular cell layer in the posterior vermis (lobules VIII and IX). It is important to determine if the cerebellum’s role in drug conditioning is a general phenomenon or is limited to a particular sensory modality.

**Methods:**

The present study evaluated the role of the posterior cerebellum (lobules VIII and IX), together with the medial prefrontal cortex (mPFC), ventral tegmental area (VTA), and nucleus accumbens (NAc) using a cocaine-induced conditioned place preference procedure with tactile cues. Cocaine CPP was tested using ascending (3, 6, 12, and 24 mg/kg) doses of cocaine in mice.

**Results:**

Compared to control groups (Unpaired and Saline animals), Paired mice were able to show a preference for the cues associated with cocaine. Increased activation (cFos expression) of the posterior cerebellum was found in cocaine CPP groups and showed a positive correlation with CPP levels. Such increases in cFos activity in the posterior cerebellum significantly correlated with cFos expression in the mPFC.

**Discussion:**

Our data suggest that the dorsal region of the cerebellum could be an important part of the network that mediates cocaine-conditioned behavior.

## 1. Introduction

Drug addiction is a learning and memory disorder characterized by escalating drug use, compulsive drug-seeking, loss of control, and a reduction of interest in other life goals and rewards (Robinson and Berridge, 2003; Edwards and Koob, 2013; Everitt, 2014). After repeated use, contexts and cues present when taking drugs can acquire incentive motivational properties of drugs of abuse. These contexts and cues anticipate drug availability and mediate instrumental behaviors promoting drug seeking and consumption even after prolonged periods of abstinence (Grant et al., 1996; Childress et al., 1999; Everitt and Robbins, 2005; Fuchs et al., 2009; Everitt, 2014; Perry et al., 2014). At a neurobiological level, it is well-known that repeated drug use induces functional and structural changes in brain circuits responsible for learning and memory processes (Robbins and Everitt, 2002; Hyman et al., 2006; Lüscher and Malenka, 2011). During the past decades, numerous studies have identified the prefrontal-limbic striatal circuitry as key for storing drug-associated memories that become dysregulated at different stages of addiction (Everitt and Wolf, 2002; Belin and Everitt, 2008; Volkow et al., 2013; Koob and Volkow, 2016).

Recent studies suggest that the cerebellum is part of the neurocircuitry that modulates brain functions altered in drug addiction (Miquel et al., 2009, 2016, 2020; Carbo-Gas et al., 2014a,b, 2017; Gil-Miravet et al., 2019, 2021). The cerebellum connects with the prefrontal cortex, striatum, amygdala, thalamus, hippocampus, and basal ganglia (Rogers et al., 2011; Bostan et al., 2013; Bostan and Strick, 2018; Gil-Miravet et al., 2019). The cerebellum sends direct (Watabe-Uchida et al., 2012; Carta et al., 2019; Gil-Miravet et al., 2021) and indirect (Forster and Blaha, 2003; Rogers et al., 2011) projections to the medial prefrontal cortex (mPFC) through the ventral tegmental area (VTA), suggesting that the VTA may be a hub by which the cerebellum regulates prefrontal and striatal function (Carta et al., 2019; Miquel et al., 2020; Gil-Miravet et al., 2021). These connections are reciprocal since the cerebellum receives inputs from the VTA (Ikai et al., 1992, 1994).

Anatomically, the cerebellum is a complex cluster of nuclei that regulate motor, cognitive and emotional functions (Schmahmann and Caplan, 2006; Glickstein et al., 2011; Van Overwalle et al., 2014; Witter and De Zeeuw, 2015; Carta et al., 2019), including the establishment of drug-conditioned memories (Carbo-Gas et al., 2014a,b; Gil-Miravet et al., 2019, 2021; Guarque-Chabrera et al., 2022a). Neuroimaging studies show that the presentation of drug-related cues leads to cerebellar activation in human addicts (Grant et al., 1996; Bonson et al., 2002; Volkow et al., 2003; Anderson et al., 2006; Moulton et al., 2014; Miquel et al., 2016). Animal cFos immunoreactivity studies have also shown that cocaine-induced conditioned preference increases neuronal expression in the vermis of the cerebellum, which strongly correlates with the level of preference for the conditioned cue in mice and rats (Carbo-Gas et al., 2014a,b). Enlarged granule cell activity results in greater stimulation onto molecular layer interneurons that in turn inhibit Purkinje cells. Therefore, because Purkinje axons send inhibitory output to the deep cerebellar nuclei (DCN) which are the major cerebellar output to other brain regions (Kebschull et al., 2020; Novello et al., 2022), we would expect a reduction of this inhibitory modulation, producing therefore the disinhibition of the DCN and subsequent cerebello-cortical loops (Miquel et al., 2020). Supporting this, Carta et al. (2019) demonstrated that optogenetic stimulation of DCN terminals in the VTA increased neuronal activity in this region. These findings, together with those from Gil-Miravet et al. (2021) suggest that the cerebellum might mediate the prefrontal-limbic-striatal system’s activation that regulates drug-related contextual memory.

Evidence demonstrating a role of the cerebellum in the conditioned effects of cocaine have so far used olfactory cues (Carbo-Gas et al., 2014a,b, 2017; Gil-Miravet et al., 2019, 2021; Guarque-Chabrera et al., 2022a). Cerebellar involvement in olfaction and olfactory stimulation-induced conditioning has been recently demonstrated (Mainland et al., 2005; Rodríguez et al., 2018; Zhang et al., 2021). Compared to visual, tactile, or auditory stimuli, olfactory cues are more effective as retrieval cues for emotional memories (Hakim et al., 2019). Also, olfactory cues are more potent than other sensory stimuli in facilitating the acquisition and retrieval of context-dependent memories (Cann and Ross, 1989; Herz, 1998; Otto et al., 2000). It is, however, necessary to explore whether the cerebellum’s role in drug-induced conditioning depends on a specific sensory modality. The use of tactile cues in conditioned place preference (CPP) is commonly used to investigate learning and memory processes underlying motivational and reinforcing effects of addictive drugs, including cocaine (for an extensive review, see Cunningham et al., 2011). Our aim in the present study was to investigate the pattern of neural activation (cFos expression) in the cerebellum, VTA, NAc, and mPFC, associated with cocaine-induced CPP expression, using a CPP procedure with tactile cues.

## 2. Materials and methods

### 2.1. Subjects

A total of 50 SWISS male mice (RjOrl:SWISS) purchased from Janvier-Labs S.A.S., (France) were 8 weeks old (30–45g) at the beginning of the study. Mice were housed 3 per cage with *ad libitum* access to food and water under controlled environmental conditions (12-h light cycle, lights on at 8:00 am). Colony rooms were maintained at 21 ± 1°C of temperature, and 50 ± 5% humidity levels. All procedures started at least a week after acclimation to the facilities. Behavioral protocols took place 2 h after lights were turned on, within the first 5 h of the light cycle. Experiments were conducted following the guidelines provided by the European Community Council Directive (2010/63/EU), Spanish directive BOE 34/11370/2013, and local directive DOGV 26/2010.

### 2.2. Drugs

We dissolved cocaine hydrochloride (Alcaliber S.A., Spain) in 0.90% saline solution at a concentration of 1 mg/mL. The drug was intraperitoneally (IP) injected (10 mL/kg) at 3, 6, 12, and 24 mg/kg doses. These doses were chosen based on previous studies demonstrating reliable CPP in mice (Itzhak and Anderson, 2012; Itzhak et al., 2013).

### 2.3. Place conditioning apparatus

The present study used six activity-monitored chambers (30 cm long × 15 cm wide × 20 cm high) contained in individual sound-attenuated and light-controlled enclosures (Cibertec S.A., Madrid, Spain). Each conditioning box was equipped with equally spaced photocells running the length of the box, 2.20 cm above the floor and 5 cm apart. These detectors were connected to a computer that recorded the motor activity counts (beam breaks expressed as cm per min) and the time each animal spent on each side of the box during all conditioning and test sessions. The boxes placed on top of two interchangeable floor halves that served as the tactile conditioned stimuli (CS). We used flooring cues (interchangeable grid and hole floors) as CS. Grid floors consisted of 2.30 mm stainless steel rods mounted 6.40 mm apart on acrylic rails. Hole floors consisted of stainless steel (16 gauge) perforated with 6.40 mm holes on 9.50 mm staggered centers, mounted on an acrylic base. We selected these floor textures based on previous studies (Cunningham et al., 2003, 2006; Font and Cunningham, 2012; Font et al., 2017).

### 2.4. General protocol

**FIGURE 1:**
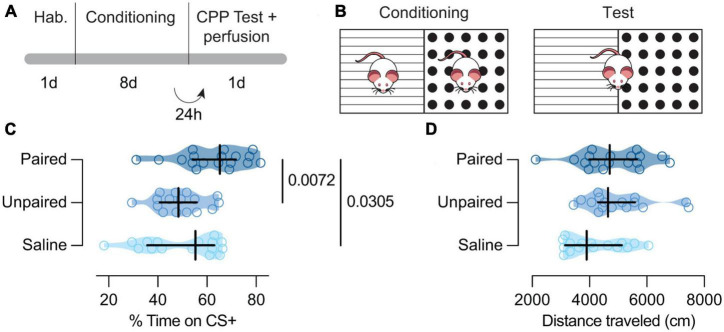
Behavioral paradigm and results. **(A)** Experiment timeline. **(B)** Schematic representation of the behavioral apparatus. **(C)** Cocaine-paired treatment encouraged a preference for cocaine-related cues.% of time spent in the CS + compartment for the Paired [dark blue; *n* = 18; *Mdn* = 65.28 (54.17, 71.18)], Unpaired [medium blue; *n* = 16; *Mdn* = 48.39 (40.79, 55.86)], and Saline [light blue; *n* = 16; *Mdn* = 55.24 (35.69, 63.01)] groups. **(D)** Cocaine-paired treatment did not affect locomotor activity on the day of the test. Distance traveled (cm) on the day of the test for the Paired [dark blue; *n* = 18; *Mdn* = 47.04 (3996.00, 5662.00)], Unpaired [medium blue; *n* = 16; *Mdn* = 4667.00 (4294.00, 5583.00)], and Saline [light blue; *n* = 16; *Mdn* = 3899.50 (3147.00, 5131.00)] groups. **(C,D)** Data are displayed using truncated violin plots and presenting individual scores (empty circles) and group scores as *Mdn* (vertical black lines) ± 95% CI (horizontal black lines).

The cocaine-induced CPP protocol involved three phases (habituation, acquisition, and test) scheduled for a period of 2 weeks (5 days a week with a 2-day weekend break) (Figure 1A). We randomly assigned mice to one of the two conditions; grid + (G +) or grid- (G-) (Cunningham et al., 2003; Figures 1A, B). Thus, half of the animals received cocaine associated with the grid floor (G +) and vehicle associated with the hole floor (H-), and the other half received cocaine associated with the hole floor (H +) and vehicle with the grid floor (G-). We also counterbalanced, for each group, the order in which each type of trial was received (drug trial first versus vehicle trial first) and the left/right position of the stimulus alternatives during testing (e.g., the grid on the left side versus the grid on the right side).

#### 2.4.1. Habituation

On day 1, mice were moved to the experimental room and allowed to rest in their home cages for 30 min (Figure 1A). After this period, mice received a saline injection and were placed immediately in the CPP apparatus. Animals were habituated to the conditioning chamber for 5 min with unrestricted access to both compartments. To avoid latent inhibition effects associated with the cues, we covered the floor of the conditioning apparatus with black cardboard paper.

#### 2.4.2. Conditioning

Before the acquisition phase (days 2–9), mice (*n* = 50) were divided into Paired (*n* = 18), Unpaired (*n* = 16), and Saline (*n* = 16) groups. Saline animals matched context exposure, whereas Unpaired mice were a control for the non-associative effect of cocaine administration. Every day, mice were transported to the experimental room, where they rested for 30 min before the session started. Based on the idea that associative strength increases when an unexpected unconditioned stimulus occurs (Rescorla and Wagner, 1972; Dunsmoor et al., 2015), some investigators have suggested that the magnitude of place conditioning would be more potent when escalating drug doses are administered to animals (Itzhak and Anderson, 2012; Conrad et al., 2013; Liddie and Itzhak, 2016). Animals in the Paired and Unpaired groups followed an ascending schedule of cocaine administration (3, 6, 12, and 24 mg/kg). The paired group received 8-conditioning sessions of 30 min each, in which the drug-paired compartment was presented on alternate days (4 days of CS +, drug-paired compartment, and 4 days of CS-, vehicle paired compartment). Unpaired animals received injections of saline or cocaine randomly associated with both floors. Saline mice received saline injections before each exposure to both tactile cues: grid and hole floors. The experimental (Paired) and control groups (Unpaired and Saline) received equal injections during conditioning. Both Paired and Unpaired groups received the same cocaine-dose number by the end of the experiment (final dose of 45 mg/kg). All injections were given immediately before placing the animal in the conditioning chamber.

#### 2.4.3. Testing

On day 10, 24 h after the last conditioning session, mice were tested for the expression of cocaine CPP (30 min) (Figures 1A, B). Mice received saline injections immediately before being placed in the middle of the CPP chamber. Time spent in the cocaine and vehicle-paired chambers was recorded. In this phase, animals choose between CS + and CS-. Preference score was calculated as the percentage of time spent on the cocaine-paired floor (CS +) as follows: {Time Spent in CS + /[(Time Spent in CS +) + (Time Spent in CS–)]} × 100. The apparatus was configured with one cue on each side of the apparatus and the left/right position of the cues was counterbalanced within each group. Since this was randomized, half of the animals received cocaine on the grid floor and the other half, on the hole floor. For the paired group, the CS + was the floor and side always associated with cocaine. During conditioning the saline and unpaired groups received their treatments associated with all the cues and positions. On the test day their CS + was determined following this distribution: for 25% of animals as hole on the right, for 25% of animals as grid on the right, for 25% of animals as hole on the left, and for 25% of animals as grid on the left. This way, both cues and positions were fully counterbalanced.

### 2.5. Perfusion Protocol and brain sampling

**FIGURE 2:**
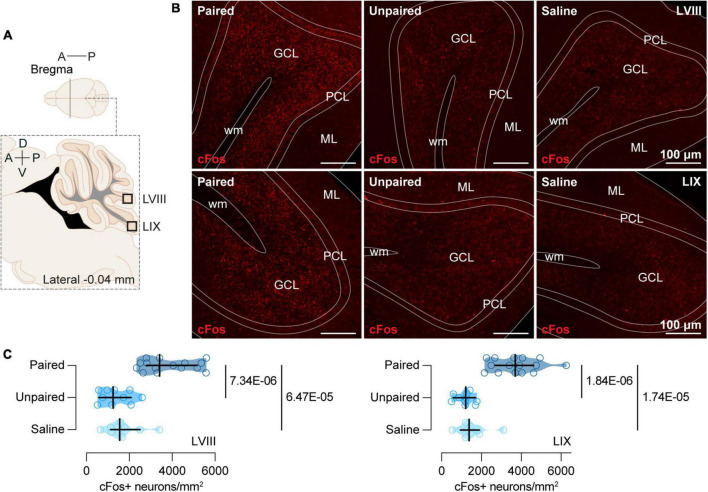
cFos expression in the posterior cerebellum. **(A)** Schematic representation of the areas of the posterior cerebellum (LVIII and LIX) where immunochemistry analyses were performed. **(B)** Representative confocal images of cFos staining in LVIII (top) and LIX (bottom) for each group (Paired, Unpaired, and Saline). ML, Molecular layer; PCL, Purkinje cell layer; GCL, Granule cells layer; wm, white matter. **(C)** Cocaine-paired treatment increased cFos expression in both LVIII and LIX of the cerebellum. Number of cFos positive cells/mm^2^ in LVIII and LIX for the Paired [dark blue; *n* = 15; LVIII: *Mdn* = 3415.31 (2799.76, 5162.68); LIX: *Mdn* = 3706.54 (2680.62, 4633.17)], Unpaired [medium blue; *n* = 10; LVIII: *Mdn* = 1250.96 (575.84, 2084.93); LIX: *Mdn* = 1211.24 (595.69, 1707.65)]), and Saline [light blue; *n* = 10; LVIII: *Mdn* = 1548.80 (1131.82, 2501.91); LIX: *Mdn* = 1389.95 (953.11, 1906.22)] groups. Data are displayed using truncated violin plots and presenting individual scores (empty circles) and group scores as *Mdn* (vertical black lines) ± 95% CI (horizontal black lines).

**FIGURE 3:**
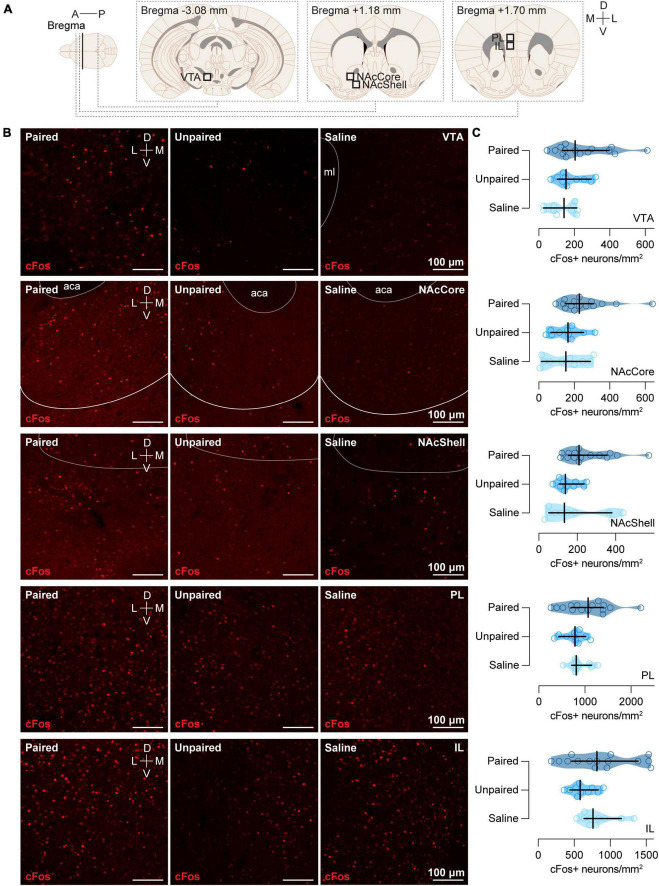
cFos expression in the VTA, NAcCore, NAcShell, PL, and IL. **(A)** Schematic representation of the areas of different brain regions (VTA, NAcCore, NAcShell, PL, and IL) where immunochemistry analyses were performed. **(B)** Representative confocal images of cFos staining in VTA, NAcCore, NAcShell, PL, and IL (top to bottom) for each group (Paired, Unpaired, and Saline). ml: medial lemniscus; aca: anterior part of the anterior commissure. **(C)** Cocaine-paired treatment did not increase cFos expression in VTA, NAcCore, NAcShell, PL, and IL. Number of cFos positive cells/mm^2^ in (top to bottom) VTA for the Paired [dark blue; *n* = 15; *Mdn* = 204.55 (134.78, 396.41)], Unpaired [medium blue; *n* = 10; *Mdn* = 153.99 (104.92, 134.78)], and Saline [light blue; *n* = 10; *Mdn* = 142.14 (27.08, 214.34)] groups; NAcCore for the Paired [dark blue; *n* = 15; *Mdn* = 228.51 (148.69, 308.34)], Unpaired [medium blue; *n* = 10; *Mdn* = 164.71 (67.69, 252.70)], and Saline (light blue; *n* = 10; *Mdn* = 152.86 [15.79, 291.06]) groups; NAcShell for the Paired [dark blue; *n* = 15; *Mdn* = 209.73 (154.95, 355.29)], Unpaired [medium blue; *n* = 10; *Mdn* = 138.76 (106.07, 234.65)], and Saline [light blue; *n* = 10; *Mdn* = 133.12 (51.89, 380.55)] groups; PL for the Paired [dark blue; *n* = 15; *Mdn* = 1068.84 (699.58, 1396.03)], Unpaired [medium blue; *n* = 10; *Mdn* = 785.36 (432.69, 1018.10)], and Saline [light blue; *n* = 10; *Mdn* = 813.90 (710.35, 1144.20)] groups; and IL for the Paired [dark blue; *n* = 15; *Mdn* = 814.87 (464.31, 1386.69)], Unpaired [medium blue; *n* = 10; *Mdn* = 582.51 (441.95, 830.67)], and Saline [light blue; *n* = 10; *Mdn* = 761.45 (638.62, 1153.46)] groups. Data are displayed using truncated violin plots and presenting individual scores (empty circles) and group scores as *Mdn* (vertical black lines) ± 95% CI (horizontal black lines).

Randomly selected subgroups of animals of each experimental group (Paired, *n* = 15; Unpaired, *n* = 10; and Saline, *n* = 10) were anesthetized with sodium pentobarbital (15 mg/kg) (Dolethal 100 mL; Vetoquinol E.V.S.A., Madrid, Spain) 90 min after the preference test and perfused transcardially, first with heparin solution (0.006%) (Heparin sodium salt from porcine intestinal mucosa; Cat# H3393; Sigma-Aldrich, Madrid, Spain) and then with paraformaldehyde (4%) (Paraformaldehyde, powder, 95%; Cat# 158127; Sigma-Aldrich, Madrid, Spain). After perfusion, each brain and cerebellum were dissected and placed in a container with the same fixative at room temperature. Twenty-four hours later, we immersed the tissue in a 30% sucrose solution in phosphate buffered (0.10 M) with sodium azide (2%) (Sodium azide BioXtra; Cat# S8032; Sigma-Aldrich, Madrid, Spain) and preserved at 4°C until the brains sunk. Then, we sliced the brain tissue into 40 μm-sections and collected four series using a cryostat microtome (Microm HM560 Thermo Fisher Scientific, Barcelona, Spain), storing them at 20°C in a cryoprotectant solution. Then, we selected sagittal sections (comprising the whole vermis) of LVIII and lobule IX (LIX) of the cerebellum (Figure 2A) and coronal sections of VTA (Figure 3A), NAc (Figure 3A), and mPFC (Figure 3A) for subsequent immunofluorescence.

### 2.6. cFos immunofluorescence

Sections were washed with phosphate-buffer saline 0.10 M triton X-100 (Sigma-Aldrich, Madrid, Spain; Cat# T9284) (1%) (PBST) and then incubated in a blocking solution (1.50% Donkey serum; Cat# D9663; Sigma-Aldrich, Madrid, Spain) in PBST for 30 min at room temperature. Subsequently, they were incubated with a polyclonal primary antibody, rabbit anti-cFos (1:500; Cat# 226003; Synaptic Systems, Göettingen, Germany) in PBST with donkey serum (1.50%), in smooth agitation at 4°C for 24 h. After washing with PBST, sections were incubated with donkey anti-rabbit Alexa Fluor 555 conjugate (1:250; Cat # A-31572; Thermo Fisher Scientific, Madrid, Spain) in PBST with donkey serum (1.50%) for 2 h at room temperature. Then, sections were rinsed with PBST and mounted with FluorSave reagent (Cat# 345789; Calbiochem; Millipore; Merck KGaA, Darmstadt, Germany).

### 2.7. Image acquisition analysis

We acquired two images per mouse and brain region using a confocal microscope at the Scientific Instrumentation Service (SCIC) of the Universitat Jaume I (Leica DMi8, Leica Microsystems CMS GmbH, Wetzlar, Germany) with 20× lenses and a resolution 1024 × 1024 px. The intensity of the 561 laser (1%), gain (750), and offset (–1) were maintained constant in each acquisition. We considered cFos +, only those cells exhibiting a uniform and consistent red labeling signal in the nucleus. We counted cFos + cells in specific regions of interest (ROI). For the granule cell layer, we established a ROI of 153.41 μm × 146.59 μm (Figures 2A, B) in sagittal sections of lobules VIII and IX of the vermis. Considering the cell size, the majority of the cFos + neurons in our study were granular cells (3–10 μm) (Sillitoe et al., 2012). We focused our assessment on LVIII and LIX because previous studies (Carbo-Gas et al., 2014a,b, 2017), demonstrated a significant correlation between preference and cFos expression in these areas. For the rest of brain regions, ROIs were determined in coronal sections of the VTA (ROI: 383.52 μm × 416.48 μm; Figures 3A, B), NAc (ROI: ROI: 383.52 μm × 416.48 μm; Figures 3A, B) and mPFC (ROI: 400.57 μm × 400.57 μm; Figures 3A, B). Cell count was performed semi-automatically with FIJI free software (Schindelin et al., 2012) using the cell counter plugin.

### 2.8. Data analysis

All data were analyzed following non-parametric analyses using GraphPad Prism 8.4.0 software (GraphPad Software, Inc., Boston, MA, USA) and R Statistical language (version 4.2.3; R Core Team, 2023). We decided to use non-parametric statistics (distribution-free methods) for two reasons. First, some of our groups did not follow a gaussian distribution under the Shapiro–Wilk or Anderson-Darling test, in the variables studied in the present research. Second, the main problem of these tests for normality is their lack of statistical power with small data sets. It is recommended to avoid parametric analyses with data samples *n* < 20, even after positive normality tests (Le Cessie et al., 2020). Non-parametric estimations make fewer assumptions about the nature of the distributions, are robust with samples *n* < 20 (our sample per group is *n* < 20), and can be used to analyze both categorical and continuous data. Given that, we used the median as a measure of central tendency and the differences between medians to calculate the magnitude of the differences between our groups. We used Kruskal–Wallis tests to determine whether our experimental groups (Paired, Unpaired, and Saline) were likely to come from the same population. When differences were demonstrated, we followed up with *post hoc* comparisons using Mann–Whitney tests. We reported effect sizes of those comparisons by using the Hodges-Lehmann difference between pairs of medians (*MndD* = *Mdn Group1- Mdn Group2*), their 95% confidence intervals (95% CI), and the exact *p*-values of those differences (Guarque-Chabrera et al., 2022b; Sanchis-Segura et al., 2022). Then, we corrected the significance level by adjusting for multiple comparisons using the False Discovery Rate method (Benjamini and Hochberg, 1995). Finally, we used Spearman’s rank correlation coefficient rho as an approach to analyze the relationship between two variables in the three experimental groups, Paired, Unpaired, and Saline. We chose this non-parametric test given that parametric statistics assume linearity on the data distribution but that is difficult to “assume” with small samples. This coefficient measures the degree of similarity between the ranks of two variables (continuous or ordinal) assessing how well their relationship can be described using a monotonic function. It requires the ranking of the data as the first step to perform the analysis, and consequently, the correlation scatter plots are displayed using the ranks to visually represent the relationship between variables. As per the interpretation, *rho* can range from –1 to 1 and its sign indicates the direction of the association; Spearman’s *rho* will be positive if both variables increase together, whereas it will be negative if one variable increases while the other decreases. Therefore, a *rho* magnitude equal to 0 would mean that one variable neither increases or decrease with the other and a *rho* magnitude of 1 would mean that both variables are perfectly monotonically associated (Spearman, 1904). To calculate Sperarman’s *rho* with its 95%CI (bootstrap, 5,000 reshuffles) and the exact *p*-value we used the functions *cor.test* of the *stats* package (R Core Team, 2023) and *spearmanRho* of the *rcompanion* package (Mangiafico, 2022). The alpha level was set at 0.05 for all statistical tests. All effects in which the confidence intervals included 0 were not considered significant even though alpha level was smaller than 0.05.

## 3. Results

### 3.1. Cocaine-Paired treatment induced preference for cocaine cues but did not affect locomotor activity

To assess whether conditioned animals with cocaine showed CPP, we performed a Kruskal–Wallis test that revealed differences in the percent of time spent in the CS +, between Paired, Unpaired, and Saline groups [*H*(2) = 9.75, *p* = 0.0076; Figure 1C]. *Post hoc* analysis showed that Paired mice expressed a higher CS + preference when compared to Unpaired [*MdnD* = 14.19 (4.85, 22.81), *p* = 0.0072; Figure 1C] and Saline treated mice [*MdnD* = 12.60 (1.72, 22.94), *p* = 0.0305; Figure 1C]. However, Unpaired and Saline animals showed similar preference for cocaine cues [*MdnD* = –1.08 (–12.89, 10.03), *p* = 0.7595; Figure 1C]. Interestingly, the locomotor activity on the test day seemed similar between our groups [*H*(2) = 5.72, *p* = 0.0572; Figure 1D].

### 3.2. Cocaine-paired treatment increased cFos expression in the posterior cerebellum but not in other regions of the brain

In both LVIII [*H*(2) = 21.96, *p* = 1.70E-05; Figure 2C] and LIX [*H*(2) = 24.09, *p* = 5.87E-05; Figure 2C] cFos was expressed differentially in our groups (Figures 2B, C). LVIII of the cerebellum showed a strong induction of cFos in the Paired group when compared to the Unpaired [*MdnD* = 2323.20 (1509.09, 3474.88), *p* = 7.34E-06; Figures 2B, C] and Saline [*MdnD* = 1965.79 (1211.24, 3117.46), *p* = 6.47E-05; Figures 2B, C] groups, whereas, Unpaired, and Saline mice showed similar cFos expression [*MdnD* = –347.49 (–953.11, 496.41), *p* = 0.4813; Figures 2B, C]. We observed a similar pattern of differences in Paired mice for the LIX. Thus, animals in the Paired group showed a greater expression of cFos than animals in the Unpaired [*MdnD* = 2488.67 (1667.94, 3243.22), *p* = 1.84E-06; Figures 2B, C] or Salline [*MdnD* = 2317.30 (1310.53, 3044.66), *p* = 1.74E-05; Figures 2B, C] groups, whereas, both Unpaired and Saline cFos expression did not differ [*MdnD* = –198.56 (–721.45, 238.28), *p* = 0.2101; Figures 2B, C].

We further assessed cFos expression in other regions of the brain. However, ascending cocaine-paired treatment did not display differences in our groups in VTA [*H*(2) = 3.81, *p* = 0.1487], NAcCore [*H*(2) = 5.01, *p* = 0.0816], NAcShell [*H*(2) = 5.17, *p* = 0.0754], Prelimbic portion of the mPFC (PL) [*H* (2) = 2.70, *p* = 0.2290], or the infralimbic portion of the mPFC (IL) [*H*(2) = 3.47, *p* = 0.1762] (Figure 3).

### 3.3. Correlation analyses

#### 3.3.1. cFos expression in the posterior cerebellum is related to the expression of preference for the CS +

**FIGURE 4:**
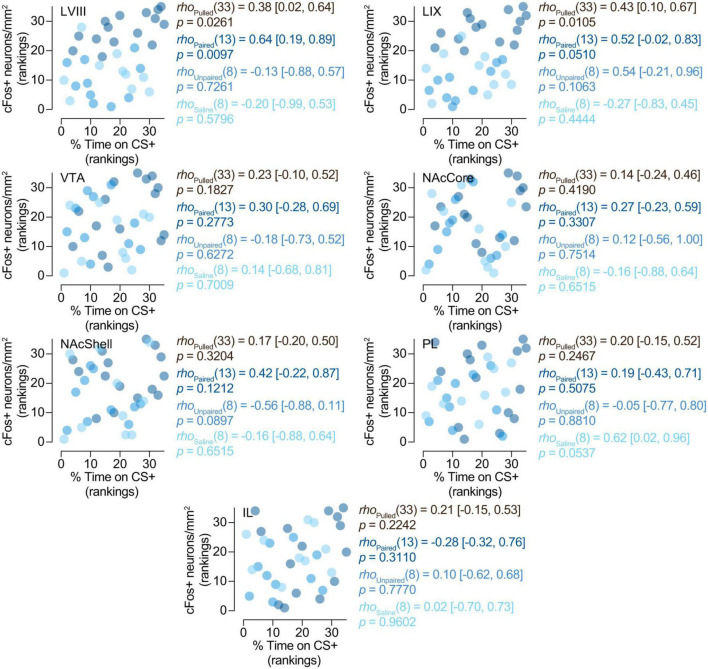
Relationship between preference and cFos expression. cFos expression in the posterior cerebellum is related to the expression of preference for the CS +. Spearman’s rank correlations (*rho* [95% CI]) between% of time on CS + and number of cFos + neurons/mm^2^ in LVIII (lobule VIII), LIX (lobule IX), VTA (Ventral tegmental area), NAcCore (Nucleus Accumbens core), NAcShell (Nucleus Accumbens shell), PL (Prelimbic cortex), and IL (Infralimbic cortex). Data is displayed using ranked scatter plots. Pulled, *n* = 35; Paired, *n* = 15; Unpaired, *n* = 10; Saline, *n* = 10.

Here we wanted to determine whether cFos expression in our regions of interest might be related to the expression of preferences for cocaine-related cues. Spearman’s rank correlation analyses yielded a positive correlation between the expression of cFos and the percentage of time spent on the CS + compartment (cocaine-paired compartment) in LVIII [*rho*(13) = 0.64 (0.19, 0.89), *p* = 0.0097] (Figure 4) for paired animals. However, we did not observe this relationship in any other region we explored (LIX, VTA, NAcCore, NAcShell, PL, and IL) or groups of treatment (paired, saline or unpaired) (Figure 4). Therefore, these results suggest that the expression of conditioned preference for cocaine cues seems to be selectively associated with increased neural activity in the apical region of the posterior cerebellar LVIII vermis.

#### 3.3.2. cFos expression is unrelated to locomotor activity on the test day

To identify whether cFos expression in our regions of interest might not only be associated with cocaine-conditioned memory but also with the locomotor activity of the day of the test, we performed Spearman’s *rho* coefficient analyses between these variables. These results revealed no relationship between cFos expression and locomotor activity on the test day between LVIII and LIX or any other evaluated areas (Table 1).

**TABLE 1:** Correlation between cFos expression in the paired group and locomotor activity on the test day.

Structure	*rho* (95% CI)	*p*-value
LVIII	–0.18 (–0.64, 0.36)	0.5041
LIX	–0.07 (–0.57, 0.47)	0.8147
VTA	0.21 (–0.36, 0.66)	0.4595
NAcCore	–0.36 (–0.75, 0.20)	0.1808
NAcShell	–0.38 (–0.76, 0.18)	0.1592
PL	–0.27 (–0.70, 0.30)	0.3282
IL	–0.49 (–0.81, 0.05)	0.0679

Results of the correlation between locomotor activity on the day of the test and the number of cFos positive cells/mm2 in LVIII, LIX, VTA, NAcCore, NAcShell, PL, and IL cortex, are displayed as Spearman’s rho and its 95% CI with the exact *p*-value (*n* = 15).

#### 3.3.3. cFos expression between LVIII and LIX and our other regions of interest

**FIGURE 5:**
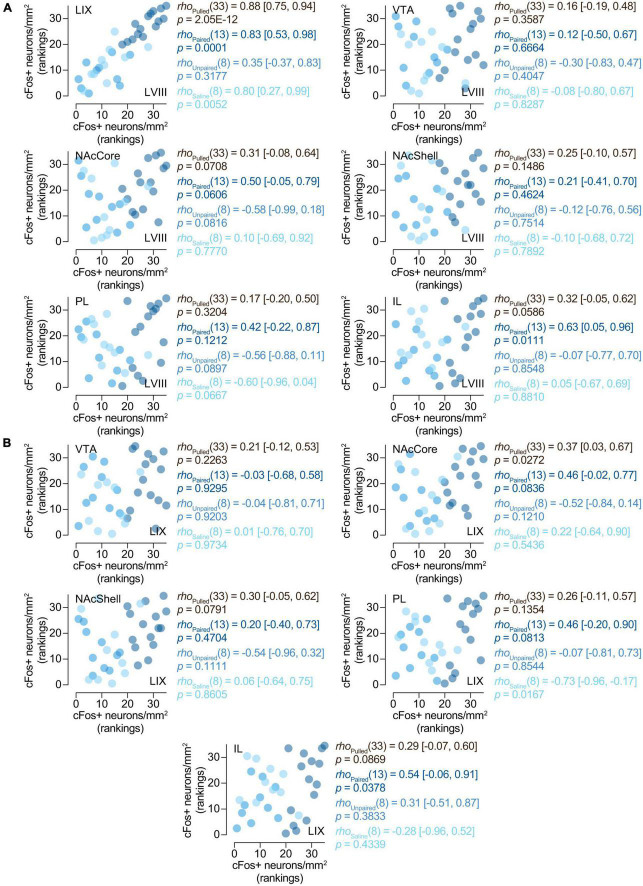
Relationships between the activity of the cerebellum and other brain region. Spearman’s rank correlations [*rho* (95% CI)] of the number of cFos + neurons/mm^2^ in **(A)** LVIII (lobule VIII), **(B)** LIX (lobule IX), and our other regions of interest, VTA (Ventral tegmental area), NAcCore (Nucleus Accumbens core), NAcShell (Nucleus Accumbens shell), PL (Prelimbic cortex), and IL (Infralimbic cortex). Data is displayed using ranked scatter plots. Pulled, *n* = 35; Paired, *n* = 15; Unpaired, *n* = 10; Saline, *n* = 10.

To assess whether the expression of cFos in the cerebellum for paired animals was associated with neural changes in any of our other brain regions of interest, we performed a series of Spearman’s rank correlations between them. Both LVIII and LIX seem to express cFos in a related manner, as shown by a positive correlation between them [*rho*(13) = 0.83 (0.53, 0.98), *p* = 0.0001; Figure 5A]. Interestingly, only LVIII exhibited a positive correlation with IL [*rho*(13) = 0.63 (0.05, 0.96), *p* = 0.0111; Figure 5A)]. No association was observed between neither lobule and NAcCore, Shell, PL, VTA (Figures 5A, B). No other concurrent cFos changes were found with any of the brain areas explored for saline or unpaired animals (Figures 5A, B).

## 4. Discussion

In the present study, we investigated the effects of cocaine-induced conditioned place preference on neural activity in the cerebellar-cortical circuit. We observed that cocaine-paired animals developed a preference for cocaine cues and displayed increased neural activity in the posterior cerebellar cortex.

Our behavioral results showed that ascending doses of cocaine produced a robust cocaine-induced CPP in SWISS mice. As shown in prior research, Unpaired mice did not show conditioned preference (Carbo-Gas et al., 2014a,b, 2017); these animals displayed indifference to the cues, ruling out the possibility that preference in the Paired group was related to non-associative factors. Previous studies suggest that changes in the magnitude of the unconditioned stimulus (i.e., ascending doses of cocaine) during conditioned associative learning may lead to differences in the strength of the conditioned response (Rescorla and Wagner, 1972; Itzhak and Anderson, 2012). CPP experiments using ascending doses of cocaine in inbred mice, such as C57/BL/6J or B6:129S F2, showed reliable preferences for the CS + (Itzhak and Anderson, 2012; Conrad et al., 2013; Liddie and Itzhak, 2016). In line with these results, our findings demonstrate that ascending doses of cocaine are also effective at inducing CPP in an outbred strain of mice.

The present immunohistochemistry findings showed increased levels of cFos expression in LVIII and LIX of the vermis in animals displaying cocaine-induced CPP. In line with our present results using tactile cues, earlier reports revealed cFos increases when measured after a cocaine-induced conditioned preference test with odor cues (Carbo-Gas et al., 2014a,b, 2017). Our data also revealed comparable levels of cFos activity in Unpaired and Saline control groups, both lower than those seen in the Paired group. These results exclude the possibility that the expression in the Paired group was independent of conditioning factors. Interestingly, Carbo-Gas et al. (2017) showed that cFos increases seen in conditioned animals were absent when animals were confined in the presence of the CS + but had no possibility of expressing the preference response. Cerebellar cortex activation, therefore, did not result from the mere presence of the conditioned cue but rather from response-selection processes driven by cue exposure. Together with previous findings, our data support a role of the cerebellum in conditioned learning expression more than in simple memory reactivation or conditioned behavioral activation. Concerning the other brain regions, we did not observe differences in cFos activity in VTA, NAc, or mPFC (when comparing Paired and control groups). Carbo-Gas et al. (2014a) found that animals that received cocaine injections followed by one of these three conditions: conditioning, random association with the stimulus (Unpaired), or absence of conditioning (Unconditioned), showed similar levels of cFos + neurons in PL and IL. Further research is needed to clarify if procedural factors (ascending vs. fixed drug dosage) or animal model differences (inbred vs. outbred mice) might account for these discrepancies between studies.

Our correlations between cFos expression and preference for the CS + are also consistent with previous data in which higher levels of preference were associated with stronger neural activation in the posterior cerebellar cortex (Carbo-Gas et al., 2014a,b; Guarque-Chabrera et al., 2022a). Although these correlational findings do not imply a causal relationship, they suggest that a paired administration of cocaine is needed to induce neural activation in the posterior cerebellum. Our data also showed that cFos immunoreactivity was not related to locomotor activity seen during testing. On test day, animals that received Paired doses of cocaine showed comparable levels of motor activity to Saline and Unpaired mice. Previous investigations have demonstrated an inverse relationship between locomotor activity and conditioned preference, showing that stronger preference in DBA/2J mice correlated with lower activity during the test session (Gremel and Cunningham, 2007). Altogether, these data suggest that increases in cFos expression in the posterior cerebellar cortex might be associated with the conditioned effects of cocaine and not with general motor activity. The present results also revealed a lack of association between cFos activity in the VTA, NAc, or mPFC and cocaine-CPP.

In our study, we also explored the relationship of cerebellar cFos activity with cFos expression found in the other areas evaluated in this study. We found a positive correlation between the activity of LVIII and LIX in the cerebellum. We also studied the relationship between VTA and the posterior cerebellum. The cerebellum and the VTA have direct (Watabe-Uchida et al., 2012; Carta et al., 2019; Gil-Miravet et al., 2021) and indirect connections (Mittleman et al., 2008; Rogers et al., 2011). The idea of a functional relationship between these two structures is also supported by data demonstrating that inputs from the cerebellum to the VTA regulate not only reward seeking (Carta et al., 2019) but also cocaine-induced CPP in rats (Gil-Miravet et al., 2021) and conditioned social preference in rodents (Carta et al., 2019). Given this evidence, it was reasonable to expect a correlation between cFos activity in the dorsal vermis and the VTA. However, this result was not found in our study. This lack of correlation does not preclude the absence of a relationship between these two areas; cFos can be useful to measure peaks of neural activation, but temporal or dynamic relationships between regions might be difficult to capture with this technique. We have previously observed that cerebellar cFos expression in rats can be optimally captured when measured 90 min after stimulus exposure (Guarque-Chabrera et al., 2022a). It could be possible that different time windows of cFos expression (mice vs. rats) could account for our lack of correlation. Future studies should explore if correlations between neural activations in these structures can be optimized when determined at different time points or if they are simply unexistent.

We found a specific correlation between cFos activity in the posterior LVIII vermis and the IL portion of the mPFC. The idea of reciprocal fronto-cerebellar networks has been previously proposed (Watson et al., 2009, 2014; Suzuki et al., 2012; Carta et al., 2019), and behavioral data seem to support this relationship (Gil-Miravet et al., 2019, 2021; Guarque-Chabrera et al., 2022a). A LVIII lesion facilitated the acquisition of cocaine-induced conditioned preference and increased neural activity in the mPFC and striatum (Gil-Miravet et al., 2019, 2021). Moreover, a selective deactivation of the IL mPFC using lidocaine facilitated the acquisition of conditioned memories associated with cocaine, accompanied by a prominent increase in neural activity of the posterior vermis (LVIII and LIX) (Gil-Miravet et al., 2019; Guarque-Chabrera et al., 2022a). These findings suggest that the cerebellum may modulate the prefrontal-limbic striatal system that regulates drug-related contextual memory (Gil-Miravet et al., 2021).

Remarkably, both increased granule cell activity and a lesion of LVIII were associated with cocaine-induced conditioned preference. In searching for an explanation, we have to consider that parallel fibers (granule cell axons) activation enhance the activity of molecular inhibitory interneurons (Dizon and Khodakhah, 2011) that in turn may decrease Purkinje inhibitory control over DCN cells. Therefore, we can expect both granule cell activation and the lesion of LVIII to facilitate cerebellar output by reducing Purkinje inhibitory control onto DCN neurons (Miquel et al., 2020).

Finally, it is important to highlight that previous studies demonstrating a role of the cerebellum in the conditioned effects of cocaine used olfactory cues (Carbo-Gas et al., 2014a,b, 2017; Gil-Miravet et al., 2019, 2021; Guarque-Chabrera et al., 2022b). Because the cerebellum has an important role in mediating olfactory stimulation and contextual conditioning induced by olfactory cues (Cann and Ross, 1989; Herz, 1998; Otto et al., 2000; Mainland et al., 2005; Rodríguez et al., 2018; Zhang et al., 2021), one possibility was that the effect would be restricted to this sensorial modality. To the best of our knowledge, our study is the first to demonstrate the importance of the cerebellum’s activity during a cocaine CPP test with tactile cues.

A role of the cerebellum beyond motor control and coordination has been extensively suggested in the last decades (Schmahmann and Caplan, 2006; Miquel et al., 2009; Van Overwalle et al., 2014; Witter and De Zeeuw, 2015; Badura et al., 2018; Deverett et al., 2018). The cerebellum mediates a wide variety of brain functions like emotional memory, reward, cognition, and language (Sacchetti et al., 2002, 2004; Turner et al., 2007; Strata et al., 2011; Adamaszek et al., 2017; Carta et al., 2019; Miquel et al., 2019; Kostadinov and Häusser, 2022; Yoshida et al., 2022). Interestingly, in recent years it has been proposed that the cerebellum would be part of distributed memory and sensorimotor networks allowing emotional and behavioral responses toward drug-related cues (Miquel et al., 2009, 2016, 2019; Moulton et al., 2014). Although our data are only correlational, this new evidence adds to the growing literature demonstrating the important role of the cerebellum in addiction. Altogether, our data support the idea that, under normal conditions, cerebellar and prefrontal circuits have a regulatory relationship (Miquel et al., 2019). However, in pathological conditions like addiction, disinhibition of different components of the striato-cortico-limbic network by the cerebellum would allow the facilitation of the establishment of cocaine-conditioned memories (Gil-Miravet et al., 2021). In this proposed mechanism (Gil-Miravet et al., 2021) the cerebellar cortex would provide an essential source of input by which emotionally salient stimuli guide cue-elicited drug-seeking behavior.

## Data availability statement

The raw data supporting the conclusions of this article will be made available by the authors, without undue reservation.

## Ethics statement

The animal study was reviewed and approved by the European Community Council Directive (2010/63/EU), Spanish directive BOE 34/11370/2013, and local directive DOGV 26/2010.

## Author contributions

OR-B: data collection, analysis, and writing parts of the original draft. LR-J: data collection and analysis. JG-C: data analysis, visualization, and review and editing. MP-B and IG-M: initial phases of data collection. RP: review and editing. MM: conceptualization, methodology, review and editing, and funding acquisition. LF: conceptualization, methodology, writing original draft, review and editing, and funding acquisition. All authors have read and approved the present version of the manuscript.
